# Effects of different VV ECMO blood flow rates on lung perfusion assessment by hypertonic saline bolus-based electrical impedance tomography

**DOI:** 10.1186/s13054-024-05055-2

**Published:** 2024-08-17

**Authors:** Hongling Zhang, Yongran Wu, Xuehui Gao, Chengchao Peng, Ruirui Li, Azhen Wang, Jiancheng Zhang, Shiying Yuan, Le Yang, Xiaojing Zou, You Shang

**Affiliations:** 1 Department of Critical Care Medicine, Union Hospital, Tongji Medical College, Huazhong University of Science and Technology, Wuhan, Hubei China; 2 Department of Emergency Medicine, Tongji Hospital, Tongji Medical College, Huazhong University of Science and Technology, Wuhan, Hubei China

**Keywords:** Veno-venous extracorporeal membrane oxygenation, Electrical impedance tomography, Perfusion, Shunt, Dead space, Ventilation/perfusion matching

## Abstract

**Objective:**

Our study aimed to investigate the effects of different extracorporeal membrane oxygenation (ECMO) blood flow rates on lung perfusion assessment using the saline bolus-based electrical impedance tomography (EIT) technique in patients on veno-venous (VV) ECMO.

**Methods:**

In this single-centered prospective physiological study, patients on VV ECMO who met the ECMO weaning criteria were assessed for lung perfusion using saline bolus-based EIT at various ECMO blood flow rates (gradually decreased from 4.5 L/min to 3.5 L/min, 2.5 L/min, 1.5 L/min, and finally to 0 L/min). Lung perfusion distribution, dead space, shunt, ventilation/perfusion matching, and recirculation fraction at different flow rates were compared.

**Results:**

Fifteen patients were included. As the ECMO blood flow rate decreased from 4.5 L/min to 0 L/min, the recirculation fraction decreased significantly. The main EIT-based findings were as follows. (1) Median lung perfusion significantly increased in region-of-interest (ROI) 2 and the ventral region [38.21 (34.93–42.16)% to 41.29 (35.32–43.75)%, *p* = 0.003, and 48.86 (45.53–58.96)% to 54.12 (45.07–61.16)%, *p* = 0.037, respectively], whereas it significantly decreased in ROI 4 and the dorsal region [7.87 (5.42–9.78)% to 6.08 (5.27–9.34)%, *p* = 0.049, and 51.14 (41.04–54.47)% to 45.88 (38.84–54.93)%, *p* = 0.037, respectively]. (2) Dead space significantly decreased, and ventilation/perfusion matching significantly increased in both the ventral and global regions. (3) No significant variations were observed in regional and global shunt.

**Conclusions:**

During VV ECMO, the ECMO blood flow rate, closely linked to recirculation fraction, could affect the accuracy of lung perfusion assessment using hypertonic saline bolus-based EIT.

**Supplementary Information:**

The online version contains supplementary material available at 10.1186/s13054-024-05055-2.

## Introduction

A hypertonic saline bolus-based electrical impedance tomography (EIT) technique has been employed to assess regional lung perfusion at the bedside [1]. This method facilitates the investigation of physiological responses to various respiratory support strategies [2–5], while also aiding in the prognostic evaluation of acute respiratory distress syndrome and the detection of acute pulmonary embolism [6, 7].

To perform this method, a hypertonic saline bolus is injected during a brief apnea, serving as a contrast agent that enhances the blood flow signal, thereby enabling clinicians to quantitatively analyze lung perfusion [1]. However, during veno-venous (VV) extracorporeal membrane oxygenation (ECMO), recirculation occurs when blood from the infusing cannula is withdrawn by the draining cannula prior to entering the pulmonary circulation [8, 9]. This phenomenon is closely associated with ECMO blood flow rate and could affect the distribution of bolus saline across the regional lung [8]. Hence, the feasibility of utilizing this method in patients on VV ECMO remains uncertain. Our study aimed to examine the effects of different ECMO blood flow rates on lung perfusion assessment using the saline bolus-based EIT technique.

## Methods

### Study design

Patients were included in this prospective, observational study conducted from March 2023 to January 2024 at the Department of Critical Care Medicine of Union Hospital, Tongji Medical College, Huazhong University of Science and Technology, Wuhan, China. The study was approved by the Institutional Research and Ethics Committee of Union Hospital (No. 2022-0851). Written informed consents were obtained from the patients’ legal representatives. The study was registered before enrollment at Clinicaltrials.gov (NCT05778292).

### Patients

The inclusion criteria were patients on VV ECMO for severe respiratory failure, endotracheal mechanical ventilation, and age ≥ 18 and ≤ 70 years. The exclusion criteria included contraindications to EIT (e.g., chest surgical wounds dressing or presence of pacemaker); refractory shock; severe cardiac dysfunction; moderate or massive pleural effusion; pulmonary embolism; severe chronic cardiopulmonary disease; body mass index > 35 kg/m^2^; and blood sodium concentration < 130 or > 145 mmol/l.

### Study protocol

Patients who met the weaning criteria according to the Extracorporeal Life Support Organization Guidelines for VV ECMO were enrolled if they fulfilled the inclusion and exclusion criteria [10]. The weaning criteria included oxygenation capacity, ventilatory reserve, PaO_2_ buffer, and response to an off-sweep gas challenge [10]. Pre-membrane blood gas analyses were performed after turning off the ECMO sweep gas for 2–3 h.

Patients were deeply sedated and paralyzed, undergoing controlled mechanical ventilation during the trial period. Before reducing ECMO blood flow, the ECMO gas flow rate was set to 2 L/min and the fraction of oxygen delivered by ECMO to 1.0. The ECMO blood flow rate was then gradually decreased from 4.5 L/min to 3.5 L/min, 2.5 L/min, 1.5 L/min, and finally to 0 L/min (by briefly clamping the ECMO blood flow circuit for saline injection), with approximately 30-min intervals between each step (Additional file 1: Fig. S1). Adjust the fraction of inspired oxygen delivered by the ventilator, without modifying other ventilator parameters, to maintain a pulse oxygen saturation of at least 97% at each ECMO blood flow rate. Data, including EIT measurements, pre- and post-membrane blood gas analyses, vital signs, and blood sodium concentrations, were collected at each ECMO blood flow step.

EIT functional images and data (detailed procedures refer to Additional file 1) were generated using the PulmoVista 500 (Dräger Medical, Lübeck, Germany). After collecting baseline data for 2 min with EIT, an end-expiratory breath hold was performed. Two seconds post-occlusion, a 10 ml bolus of 5% NaCl solution was rapidly injected within 2 s through the central venous catheter. Ventilated and perfused regions were defined as pixels exceeding 10% of the maximum on the functional ventilation and perfusion maps, respectively. For quantitative analysis, the lungs were sub-segmented into ventral and dorsal regions, defined from ventral to dorsal as region-of-interest (ROI) 1, ROI 2, ROI 3, and ROI 4.

The recirculation fraction within the ECMO circuit was estimated using the SvO_2_ method [8] (Additional file 1).

### Study outcomes

The primary outcome was regional perfusion distribution at each ECMO blood flow rate. The secondary outcomes included ventilation/perfusion (V/Q) matching, dead space, shunt, and recirculation fraction at each ECMO blood flow rate.

### Statistical analysis

A formal sample size calculation was not performed and, similar to prior physiological research, 15 patients were enrolled [2, 3, 11, 12]. Categorical data were summarized as frequencies and proportions. Continuous data were presented as medians (25–75th) or means ± standard deviations according to the normality of data checked by the Shapiro–Wilk test. To compare the data obtained at each ECMO blood flow rate, either repeated measures ANOVA or the Friedman test was used, with subsequent post-hoc Bonferroni’s or Dunn’s multiple comparisons applied as appropriate. Nonparametric Spearman correlation analysis was employed to assess the correlation between various ECMO flow rates and recirculation fractions. A two-tailed *p* value of less than 0.05 was considered statistically significant. Statistical analyses were computed with GraphPad Prism 9.0 (GraphPad Software, San Diego, CA, USA).

## Results

Fifteen patients (13 male, 2 female) were included in the study. All patients were treated with femoro-jugular VV ECMO. More detailed clinical characteristics are available in Additional file 1: Table S1. No significant variations in heart rate, mean arterial pressure, pulse oxygen saturation, or blood sodium ion concentration were observed corresponding to reductions in ECMO blood flow (Additional file 1: Table S2). Higher ECMO blood flow rate was associated with higher recirculation fraction (Additional file 1: Fig. S2).

**Fig. 1:**
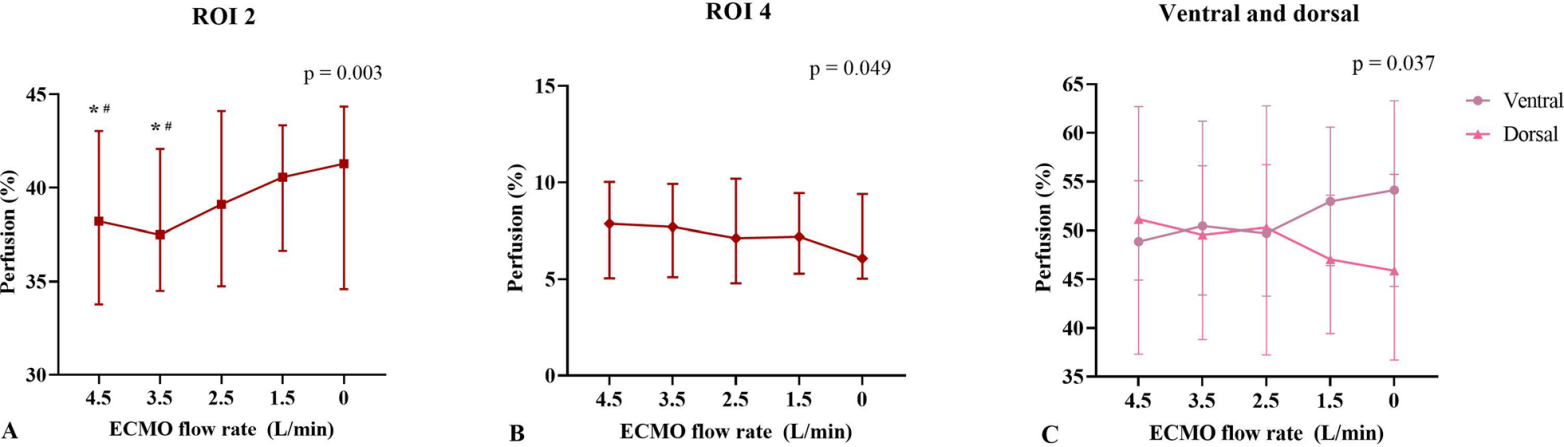
Perfusion evolution in ROI 2 (**A**), ROI 4 (**B**), and ventral and dorsal regions (**C**) in response to the decrease in ECMO blood flow rates. *ECMO* Extracorporeal membrane oxygenation, *ROI* Region of Interest. * Versus ECMO blood flow 0 L/min, *p* < 0.05, ^#^ Versus ECMO blood flow 1.5 L/min, *p* < 0.05

**Fig. 2:**
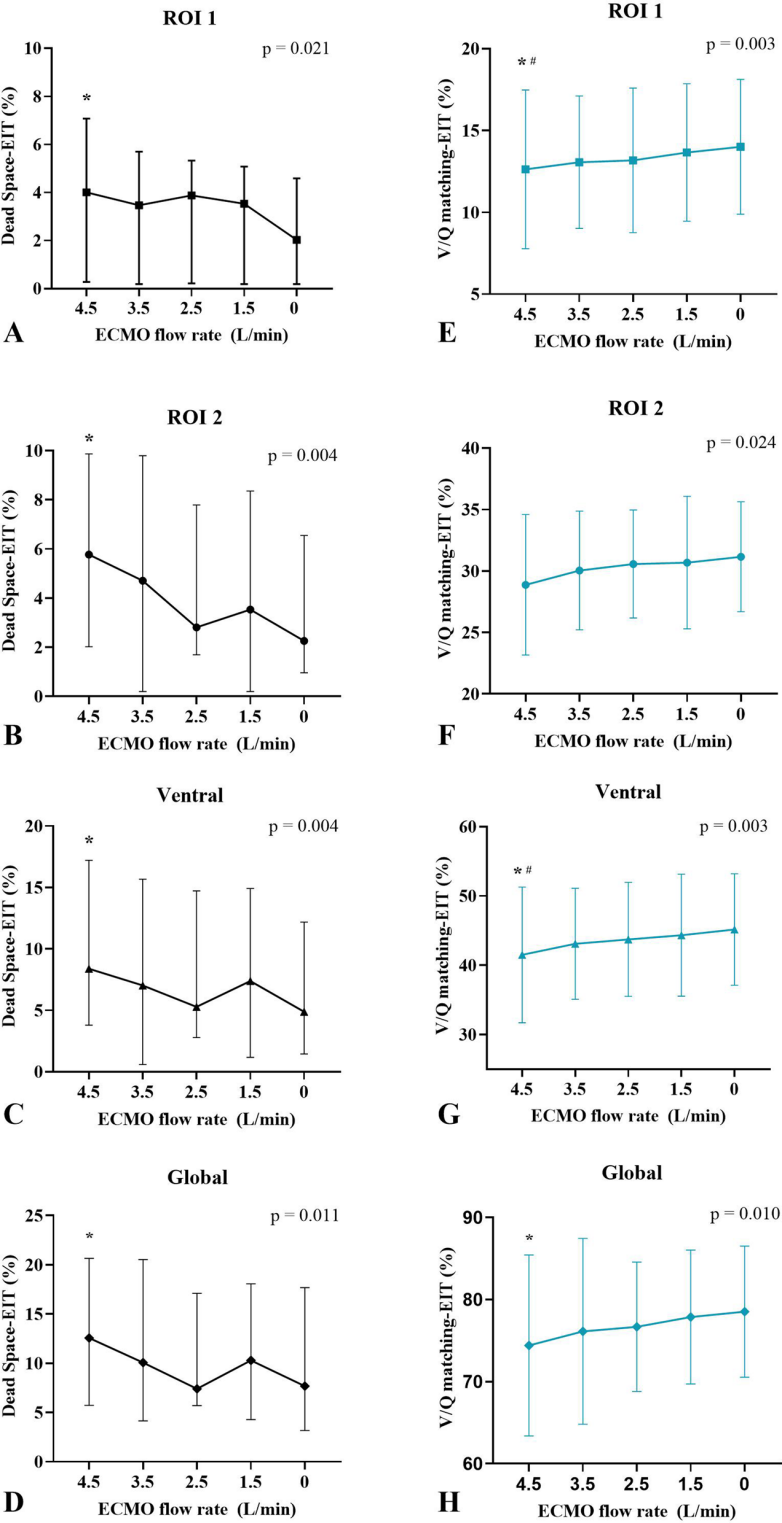
Dead space evolution in ROI 1 (**A**), ROI 2 (**B**), ventral region (**C**) and global region (**D**) in response to the decrease in ECMO blood flow rates. Ventilation/perfusion matching evolution in ROI 1 (**E**), ROI 2 (**F**), ventral region (**G**) and global region (**H**) following the decrease in ECMO blood flow rates. *ECMO* Extracorporeal membrane oxygenation, *ROI* Region of Interest. * Versus ECMO blood flow 0 L/min, *p* < 0.05, ^#^ Versus ECMO blood flow 1.5 L/min, *p* < 0.05

EIT-derived data revealed that median lung perfusion in ROI 2 and the ventral region significantly increased [38.21 (34.93–42.16)% to 41.29 (35.32–43.75)%, *p* = 0.003, and 48.86 (45.53–58.96)% to 54.12 (45.07–61.16)%, *p* = 0.037, respectively), whereas in ROI 4 and dorsal region, it significantly decreased [7.87 (5.42–9.78)% to 6.08 (5.27–9.34)%, *p* = 0.049, and 51.14 (41.04–54.47)% to 45.88 (38.84–54.93)%, *p* = 0.037, respectively] with the decrement of ECMO blood flow from 4.5 L/min to 0 L/min (Fig. 1). Dead space decreased significantly, whereas V/Q matching increased significantly in ROI 1, ROI 2, ventral, and global regions as ECMO blood flow decreased (Fig. 2). No significant alterations in regional lung ventilation distribution, end-expiratory lung impedance, or regional and global shunt were observed following reductions in ECMO blood flow. (Additional file 1: Table S2).

## Discussion

To the best of our knowledge, this is the first study to investigate the effects of different ECMO blood flow rates on hypertonic saline bolus-based EIT assessment for lung perfusion in patients on VV ECMO.

Our study demonstrates that as ECMO flow rates decrease from 4.5 L/min to 0 L/min, the fraction of lung perfusion distribution measured by EIT significantly increases in ROI 2 and the ventral region, while decreasing in ROI 4 and the dorsal region. Specifically, there were no significant alterations in the measured fraction of lung perfusion distribution with ECMO blood flow rate of 2.5 L/min or less, corresponding to a median recirculation fraction of no more than 23%. However, caution is suggested when interpreting these results since the recirculation fraction is influenced not only by ECMO blood flow but also by cannula position, volume status, and cardiac function [8, 9]. Higher ECMO flow rates, which correspond to higher recirculation fractions, could have a stronger influence on the distribution of bolus saline across the regional lung, affecting the accuracy of lung perfusion assessment by saline bolus-based EIT method.

When perfusion is integrated with ventilation, it provides a quantification of shunt (unventilated regions), dead space (unperfused regions), and V/Q matching [1]. Our study further demonstrates that changes in ECMO flow rates do not affect monitored shunt but have a significant impact on monitored dead space and V/Q matching. This observation may be attributed to the alterations in the distribution of bolus saline throughout the regional lung during decreased ECMO flow rates, with no changes detected in lung ventilation as assessed by end-expiratory lung impedance and ventilation distribution.

Our study has several limitations. First, cardiac output, which might influence monitored lung perfusion, was not assessed in this cohort. However, considering the short study duration, the patients being deeply sedated and paralyzed, the stable circulatory conditions, and the negligible effect of VV ECMO blood flow on systemic circulation [10, 13], it is assumed that the cardiac output remained constant during the trial period. Second, we did not perform repeated measurements at each blood flow rate to reduce experimental error. Given concerns about the effects of brief, high-volume infusions of hypertonic saline on blood osmolarity, we limited our measurements to one per flow rate. Third, shunt and dead space regions were defined as pixels less than 10% of the maximum of the EIT functional ventilation and perfusion maps. Variations in pixel threshold definitions for shunt and dead space in EIT software analysis might influence the study outcomes. Nonetheless, this threshold is widely adopted in current research [2, 5, 6]. Fourth, the sample size was limited.

## Conclusions

During VV ECMO, the ECMO blood flow rate, closely linked to recirculation fraction, could affect the accuracy of lung perfusion assessment using hypertonic saline bolus-based EIT.

### Supplementary Information

Additional file1

## Data Availability

The datasets used and/or analyzed during the current study are available from the corresponding author on reasonable request.

## Abbreviations

VV: Veno-venous
ECMO: Extracorporeal membrane oxygenation
EIT: Electrical impedance tomography
V/Q: Ventilation/perfusion
ROI: Region-of-interest

## References

[1] Franchineau G, Jonkman AH, Piquilloud L, Yoshida T, Costa E, Rozé H, et al. Electrical impedance tomography to monitor hypoxemic respiratory failure. Am J Respir Crit Care Med. 2023;rccm.202306-1118CI. 10.1164/rccm.202306-1118CI 38127779

[2] Wang Y, Zhong M, Dong M, Song J, Zheng Y, Wu W, et al. Prone positioning improves ventilation–perfusion matching assessed by electrical impedance tomography in patients with ARDS: a prospective physiological study. Crit Care. 2022;26:154. 35624489 10.1186/s13054-022-04021-0 PMC9137443

[3] Li R, Wu Y, Zhang H, Wang A, Zhao X, Yuan S, et al. Effects of airway pressure release ventilation on lung physiology assessed by electrical impedance tomography in patients with early moderate-to-severe ARDS. Crit Care. 2023;27:178. 37158961 10.1186/s13054-023-04469-8 PMC10169478

[4] Pavlovsky B, Pesenti A, Spinelli E, Scaramuzzo G, Marongiu I, Tagliabue P, et al. Effects of PEEP on regional ventilation-perfusion mismatch in the acute respiratory distress syndrome. Crit Care. 2022;26:211. 35818077 10.1186/s13054-022-04085-y PMC9272883

[5] Fossali T, Pavlovsky B, Ottolina D, Colombo R, Basile MC, Castelli A, et al. Effects of prone position on lung recruitment and ventilation-perfusion matching in patients with COVID-19 acute respiratory distress syndrome: a combined CT scan/electrical impedance tomography study. Crit Care Med. 2022;50:723–32. 35200194 10.1097/CCM.0000000000005450 PMC9005091

[6] Spinelli E, Kircher M, Stender B, Ottaviani I, Basile MC, Marongiu I, et al. Unmatched ventilation and perfusion measured by electrical impedance tomography predicts the outcome of ARDS. Crit Care. 2021;25:192. 34082795 10.1186/s13054-021-03615-4 PMC8173510

[7] He H, Chi Y, Long Y, Yuan S, Zhang R, Frerichs I, et al. Bedside evaluation of pulmonary embolism by saline contrast electrical impedance tomography method: a prospective observational study. Am J Respir Crit Care Med. 2020;202:1464–8. 32585116 10.1164/rccm.202005-1780LE PMC7667910

[8] Abrams D, Bacchetta M, Brodie D. Recirculation in venovenous extracorporeal membrane oxygenation. ASAIO J. 2015;61:115–21. 25423117 10.1097/MAT.0000000000000179

[9] Xie A, Yan TD, Forrest P. Recirculation in venovenous extracorporeal membrane oxygenation. J Crit Care. 2016;36:107–10. 27546757 10.1016/j.jcrc.2016.05.027

[10] Tonna JE, Abrams D, Brodie D, Greenwood JC, Rubio Mateo-Sidron JA, Usman A, et al. Management of adult patients supported with venovenous extracorporeal membrane oxygenation (VV ECMO): guideline from the extracorporeal life support organization (ELSO). ASAIO J. 2021;67:601–10. 33965970 10.1097/MAT.0000000000001432 PMC8315725

[11] Lytra E, Kokkoris S, Poularas I, Filippiadis D, Cokkinos D, Exarhos D, et al. The effect of high-flow oxygen via tracheostomy on respiratory pattern and diaphragmatic function in patients with prolonged mechanical ventilation: a randomized, physiological, crossover study. J Intensive Med. 2024;4:202–8. 38681788 10.1016/j.jointm.2023.11.008 PMC11043636

[12] Menga LS, Delle Cese L, Rosà T, Cesarano M, Scarascia R, Michi T, et al. Respective effects of helmet pressure support, continuous positive airway pressure, and nasal high-flow in hypoxemic respiratory failure: a randomized crossover clinical trial. Am J Respir Crit Care Med. 2023;207:1310–23. 36378814 10.1164/rccm.202204-0629OC PMC10595442

[13] Russ M, Steiner E, Boemke W, Busch T, Melzer-Gartzke C, Taher M, et al. Extracorporeal membrane oxygenation blood flow and blood recirculation compromise thermodilution-based measurements of cardiac output. ASAIO J. 2022;68:721–9. 34860710 10.1097/MAT.0000000000001592 PMC9067097

