# Supplementary material for: Effects of different VV ECMO blood flow rates on lung perfusion assessment by hypertonic saline bolus-based electrical impedance tomography
Source: Crit Care. 2024 Aug 17;28:274. doi: 10.1186/s13054-024-05055-2 (PMC11330074; doi:10.1186/s13054-024-05055-2)
Supplement: Supplementary file 1 — Additional file1 [file 13054_2024_5055_MOESM1_ESM.docx]

**Additional file 1**

**Effects of different VV ECMO blood flow rates on lung perfusion assessment by hypertonic saline bolus-based electrical impedance tomography**

Hongling Zhang, Yongran Wu, Xuehui Gao, Chengchao Peng, Ruirui Li, Azhen Wang, Jiancheng Zhang, Shiying Yuan, Le Yang, Xiaojing Zou, You Shang

**Methods**

*EIT assessment methods*

EIT (electrical impedance tomography) functional images and data were generated by PulmoVista 500 (Dräger Medical, Lübeck, Germany). The EIT belt with 16 surface electrodes was placed around the thorax at the fourth intercostal space level. EIT images were continuously recorded at 20 Hz. After a baseline recording of EIT data for 2 min, we performed an end-expiratory breath hold lasting 10 s. Two seconds after the start of the occlusion, a bolus of 10 ml of 5% NaCl solution was manually injected via the central venous catheter. The bolus of saline solution, injected in less than 2 s, passed through the pulmonary circulation producing an impedance dilution curve that follows typical first-pass kinetics [1,2]. To ensure accuracy in measuring end-expiratory lung impedance (EELI), the belt remained secured during the study period [3]. Patients were positioned in a semi-recumbent position with a 15°bed incline during EIT measurements [4].

EIT ventilation maps were analyzed offline to average the values of five consecutive respiratory cycles. For the quantitative analysis of the ventilation and perfusion distributions by EIT, the lungs were sub-segmented into ventral and dorsal regions. Ventral regions included two regions-of-interest (ROIs): ROI 1 and ROI 2 (mid-ventral). Dorsal region included two ROIs: ROI 3 (mid-dorsal) and ROI.

Regional functional ventilation map was calculated by subtracting the end-expiration impedance from the end-inspiration impedance, which represents the local volume variation during tidal breathing. From the analysis of ventilation maps, we assessed the following measures:

1. The percentage of ventilation distribution in the respective region.
2. EELI, corresponding to the impedance at the end of expiration.

Regional functional perfusion maps were derived from analyzing the slope of time-impedance curve after saline injection after removing the cardiac region from the images. Ventilated and perfused regions were defined as pixels higher than 10% maximum of the functional ventilation and perfusion maps, respectively. From the analysis of perfusion maps, four measures were calculated:

1. The percentage of perfusion distribution in the respective ROIs.

2. Dead space (%), corresponding to regions that were only ventilated.

3. Shunt (%), corresponding to regions that were only perfused.

4. V/Q matching (%), corresponding to regions that were both ventilated and perfused.

*Method for estimating fraction of recirculation within the ECMO circuit*

The fraction of recirculation within the ECMO circuit was estimated using SvO_2_ method with the following equation [5].

Recirculation (%) = (SpreO_2_ - SvO_2_)/ (SpostO_2_ - SvO_2_) × 100%

Where SvO_2_ represents the saturation of blood drawn from the patient via the venous drainage cannula, obtained after turning off the ECMO sweep gas for 2-3 hours. SpreO_2_ is the saturation of blood entering the oxygenator, SpostO_2_ is the saturation of blood leaving the oxygenator.

Table S1 Patients' main characteristics

| Variables | All Patients, n = 15 |
| --- | --- |
| Patients' characteristics |  |
| Male, n (%) | 13 (86.7) |
| Age (years) | 58 (34–68) |
| Body mass index (kg/m^2^) | 22.5 (19.0–26.8) |
| Comorbidities, n (%) |  |
| Hypertension | 4 (26.7) |
| Diabetes | 3 (20.0) |
| Etiology for respiratory failure, n (%) |  |
| Pneumonia | 12 (80.0) |
| Severe acute pancreatitis | 3 (20.0) |
| SOFA score at enrollment | 8 (5–10) |
| MV duration before ECMO (days) | 4 (1–9) |
| ECMO duration (days) | 10 (7–16) |
| Respiratroy parameters before weaning of ECMO |  |
| FiO_2_ by ventilator (%) | 40.0 (40.0–50.0) |
| PH | 7.41 (7.36–7.45) |
| PaO_2_/FiO_2_ (mmHg) | 225.0 (201.0–245.0) |
| PaCO_2_ (mmHg) | 40.1 (35.9–42.7) |
| Static respiratory system compliance (mL/cmH_2_O) | 30.8 (25.0–37.8) |
| Positive end-expiratory pressure (cm H_2_O) | 10.0 (8.0–10.0) |
| Tidal volume (mL) | 400.0 (360.0–450.0) |
| Tidal volume (mL/kg predicted body weight) | 6.6 (5.9–7.0) |
| Respiratory rate (cycles/min) | 20.0 (18.0–25.0) |
| Minute ventilation (L/min) | 8.20 (7.09–9.90) |

SOFA, sequential organ failure assessment; MV, mechanical ventilation; FiO_2_, fraction of inspired oxygen; PaO_2_, partial pressure of arterial oxygen; PaCO_2_, partial pressure of carbon dioxide

Table S2 Electrical impedance tomography data analysis of selected physiologic variables, fraction of recirculation, vital signs, and blood sodium concentrations at each ECMO blood flow rate.

| Variables | ECMO blood flow  4.5 L/min | ECMO blood flow  3.5 L/min | ECMO blood flow  2.5 L/min | ECMO blood flow  1.5 L/min | ECMO blood flow  0 L/min | *P* value |
| --- | --- | --- | --- | --- | --- | --- |
| EIT data |  |  |  |  |  |  |
| Perfusion distribution,  ROI 1 (%) | 10.88 [9.33–16.24] | 11.46 [10.09–16.34] | 11.58 [8.85–16.36] | 11.60 [9.86–16.12] | 12.83 [9.97–15.93] | 0.682 |
| Perfusion distribution,  ROI 2 (%) | 38.21 [34.93–42.16] ^*#^ | 37.50 [35.01–41.93] ^*#^ | 39.12 [35.70–43.45] | 40.57 [37.01–43.26] | 41.29 [35.32–43.75] | 0.003 |
| Perfusion distribution,  ROI 3 (%) | 43.27 [36.15–44.59] | 42.46 [34.42–44.63] | 42.11 [33.96–45.18] | 41.75 [34.99–45.13] | 39.81 [34.26–42.74] | 0.203 |
| Perfusion distribution,  ROI 4 (%) | 7.87 [5.42–9.78] | 7.71 [5.57–9.58] | 7.13 [4.98–9.39] | 7.20 [5.54–9.14] | 6.08 [5.27–9.34] | 0.049 |
| Perfusion distribution, ventral (%) | 48.86 [45.53–58.96] | 50.45 [43.45–60.63] | 49.71 [43.83–61.35] | 52.96 [46.76–60.51] | 54.12 [45.07–61.16] | 0.037 |
| Perfusion distribution, dorsal (%) | 51.14 [41.04–54.47] | 49.55 [39.37–56.55] | 50.29 [38.65–56.17] | 47.04 [39.49–53.24] | 45.88 [38.84–54.93] | 0.037 |
| Ventilation distribution, ROI 1 (%) | 15.03 [13.61–18.99] | 14.97 [13.47–18.84] | 15.45 [13.51–18.88] | 15.29 [13.33–18.30] | 15.29 [13.58–18.61] | 0.185 |
| Ventilation distribution, ROI 2 (%) | 46.91 [42.18–54.92] | 46.99 [42.27–54.68] | 46.51 [42.98–54.01] | 45.95 [42.92–54.50] | 46.13 [42.93–54.78] | 0.809 |
| Ventilation distribution, ROI 3 (%) | 31.04 [22.95–38.39] | 30.51 [23.36–38.14] | 30.64 [23.82–38.02] | 30.80 [24.04–38.13] | 30.59 [23.95–38.22] | 0.161 |
| Ventilation distribution, ROI 4 (%) | 6.29 [4.31–7.98] | 6.18 [4.41–7.83] | 6.01 [3.91–7.45] | 5.70 [3.97–7.99] | 6.38 [3.99–7.77] | 0.355 |
| Ventilation distribution, ventral (%) | 63.19 [57.14–72.98] | 63.35 [57.08–72.58] | 64.31 [57.04–72.53] | 63.28 [56.84–72.19] | 62.80 [56.90–72.30] | 0.535 |
| Ventilation distribution, dorsal (%) | 36.81 [27.02–42.86] | 36.65 [27.42–42.92] | 35.69 [27.47–42.96] | 36.72 [27.81–43.16] | 37.20 [27.70–43.10] | 0.535 |
| Shunt, ROI 1 (%) | 1.35 [0.94–2.39] | 1.37 [0.55–2.24] | 1.30 [0.78–2.71] | 1.84 [1.02–2.10] | 1.94 [0.88–2.50] | 0.697 |
| Shunt, ROI 2 (%) | 1.17 [0.23–2.03] | 0.91 [0.49–2.12] | 1.17 [0.30–1.70] | 1.17 [0.39–2.58] | 0.96 [0.35–1.88] | 0.521 |
| Shunt, ROI 3 (%) | 5.38 [3.02–9.14] | 6.85 [1.90–9.13] | 6.16 [2.82–8.93] | 4.52 [2.25–8.88] | 4.33 [3.15–9.93] | 0.527 |
| Shunt, ROI 4 (%) | 2.33 [0.75–4.26] | 2.33 [0.79–2.66] | 2.52 [0.92–3.32] | 2.59 [0.26–3.08] | 2.11 [0.42–3.63] | 0.254 |
| Shunt, ventral (%) | 2.75 [1.90–3.99] | 2.65 [1.69–3.73] | 2.80 [1.96–3.83] | 3.13 [2.03–4.30] | 3.65 [1.54–3.91] | 0.569 |
| Shunt, dorsal (%) | 9.32 [5.94–11.22] | 9.01 [5.24–11.36] | 8.56 [6.21–10.26] | 7.63 [5.26–10.78] | 7.28 [5.79–10.59] | 0.510 |
| Shunt, global (%) | 12.30 [9.43–14.27] | 12.10 [8.38–13.73] | 11.70 [8.52–14.96] | 11.29 [8.80–14.16] | 11.26 [7.57–14.48] | 0.969 |
| Dead space, ROI 1 (%) | 4.01 [0.37–6.67] ^*^ | 3.47 [0.21–5.66] | 3.88 [0.35–5.26] | 3.53 [0.29–5.06] | 2.03 [0.14–4.58] | 0.021 |
| Dead space, ROI 2 (%) | 5.77 [2.27–8.98] ^*^ | 4.71 [0.20–8.78] | 2.80 [1.90–6.77] | 3.53 [0.52–7.39] | 2.26 [1.20–5.58] | 0.004 |
| Dead space, ROI 3 (%) | 1.14 [0.33–2.54] | 0.94 [0.42–2.19] | 0.93 [0.11–4.03] | 1.19 [0.49–2.49] | 0.95 [0.22–2.87] | 0.778 |
| Dead space, ROI 4 (%) | 1.83 [0.82–2.88] | 1.56 [0.82–3.38] | 1.85 [0.78–3.61] | 1.51 [0.48–2.67] | 2.13 [0.45–3.08] | 0.709 |
| Dead space, ventral (%) | 8.38 [3.89–15.21] ^*^ | 7.03 [1.27–14.23] | 5.28 [3.07–13.02] | 7.38 [1.18–12.48] | 4.88 [2.02–10.66] | 0.004 |
| Dead space, dorsal (%) | 3.27 [1.49–5.79] | 2.81 [0.94–6.30] | 3.65 [1.61–5.50] | 3.57 [1.56–4.71] | 3.15 [1.43–5.86] | 0.582 |
| Dead space, global (%) | 12.57 [7.12–18.36] ^*^ | 10.07 [4.64–17.65] | 7.41 [6.00–16.79] | 10.28 [4.46–15.91] | 7.67 [3.76–15.59] | 0.011 |
| V/Q matching, ROI 1 (%) | 12.63 ± 4.85 ^*#^ | 13.06 ± 4.05 | 13.17 ± 4.41 | 13.66 ± 4.20 | 14.00 ± 4.12 | 0.003 |
| V/Q matching, ROI 2 (%) | 28.88 ± 5.72 | 30.04 ± 4.83 | 30.57 ± 4.40 | 30.69 ± 5.40 | 31.16 ± 4.48 | 0.024 |
| V/Q matching, ROI 3 (%) | 26.42 ± 4.64 | 26.27 ± 5.60 | 26.58 ± 4.45 | 26.75 ± 4.54 | 26.86 ± 4.20 | 0.788 |
| V/Q matching, ROI 4 (%) | 6.48 ± 4.65 | 6.75 ± 4.75 | 6.36 ± 4.72 | 6.77 ± 4.60 | 6.50 ± 4.95 | 0.392 |
| V/Q matching, ventral (%) | 41.50 ± 9.79 ^*#^ | 43.10 ± 8.00 | 43.74 ± 8.21 | 44.34 ± 8.81 | 45.16 ± 8.04 | 0.003 |
| V/Q matching, dorsal (%) | 32.89 ± 8.48 | 33.02 ± 9.36 | 32.94 ± 8.60 | 33.52 ± 8.38 | 33.35 ± 8.42 | 0.801 |
| V/Q matching, global (%) | 74.40 ± 11.02 ^*^ | 76.12 ± 11.32 | 76.68 ± 7.88 | 77.87 ± 8.16 | 78.51 ± 7.99 | 0.010 |
| EELI | 1300 ± 549.6 | 1289 ± 554.0 | 1301 ± 547.6 | 1292 ± 533.6 | 1302 ± 555.1 | 0.691 |
| Fraction of recirculation (%) | 39.3 [31.82–52.61] ^#✝^ | 30.77 [22.73–42.5] ^#^ | 23.08 [17.63–34.47] | 12.38 [6.34–20.45] | NA | <0.001 |
| Heart rate (cycles/min) | 101.0 [76.0–102.0] | 86.0 [76.0–106.0] | 92.0 [80.0–108.0] | 98.0 [80.0–106.0] | 99.0 [79.0–106.0] | 0.252 |
| Mean arterial pressure (mmHg) | 84.7 [77.7–101.3] | 85.0 [78.3–96.0] | 83.7 [80.7–90.0] | 84.0 [78.3–92.7] | 82.3 [78.7–98.3] | 0.470 |
| Pulse oxygen saturation (%) | 100.0 [100.0–100.0] | 100.0 [99.0–100.0] | 100.0 [99.0–100.0] | 100.0 [99.0–100.0] | 100.0 [99.0–100.0] | 0.106 |
| Na^+^ (mmol/l) | 137.9 ± 2.83 | 137.9 ± 2.72 | 138.3 ± 2.89 | 138.4 ± 2.72 | 138.2 ± 2.86 | 0.072 |

Data are median (interquartile range) or mean ± SD

ROI, region-of-interest; V/Q, ventilation/perfusion; EELI, end-expiratory lung impedance; Na^+^, blood sodium ion concentration; NA, not applicable

^*^ vs. ECMO blood flow 0 L/min, *p*< 0.05

^#^ vs. ECMO blood flow 1.5 L/min, *p*< 0.05

^✝^ vs. ECMO blood flow 2.5 L/min, *p* < 0.05


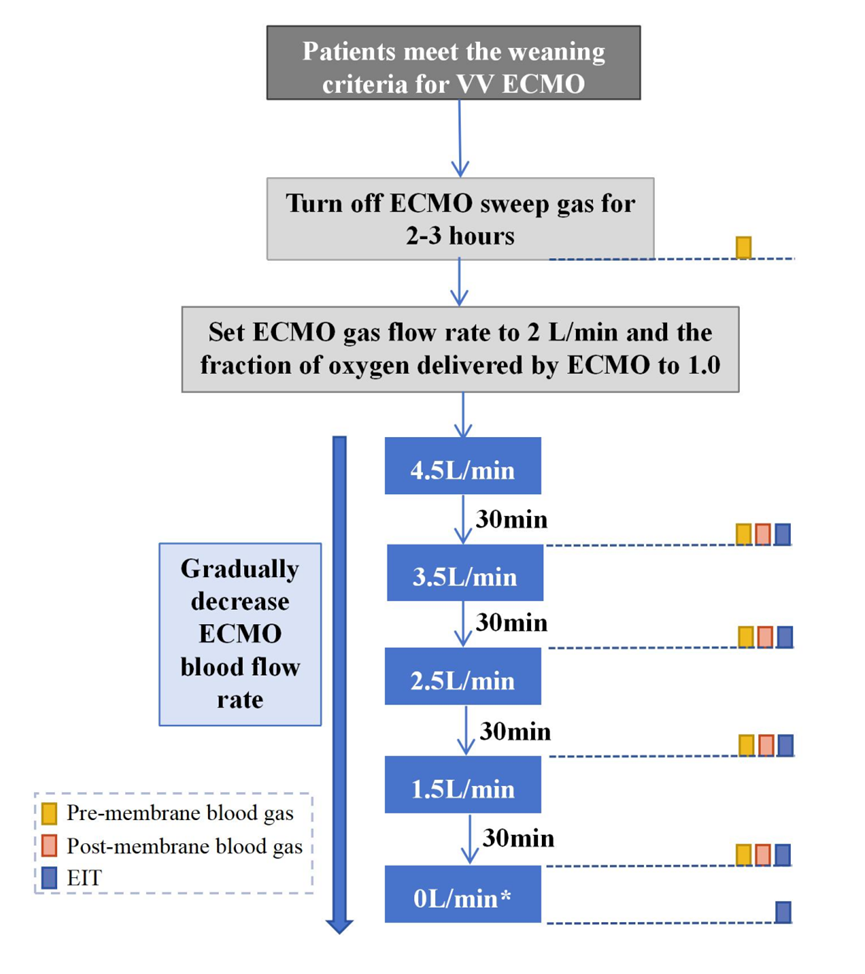


Fig.S1 Flow diagram of the trial

* Briefly clamp the ECMO blood flow circuit during saline injection

ECMO, extracorporeal membrane oxygenation; EIT, electrical impedance tomography


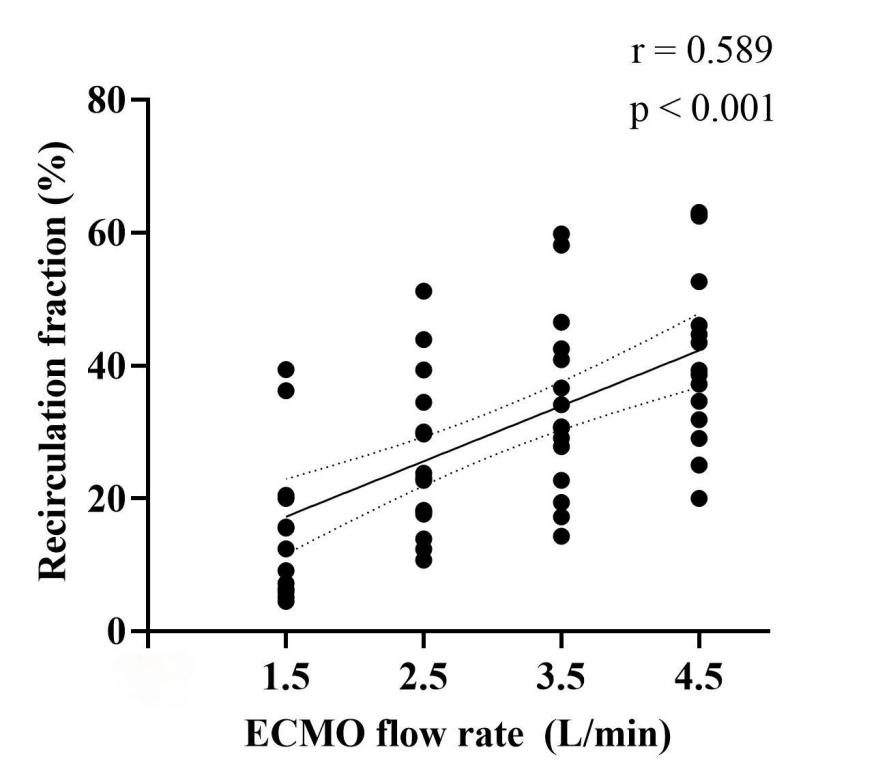


Fig.S2 Correlation between ECMO flow rate and recirculation fraction in patients on VV ECMO. Higher ECMO blood flow rate was associated with higher recirculation fraction (r = 0.589, p < 0.001).

**References**

1. Fossali T, Pavlovsky B, Ottolina D, Colombo R, Basile MC, Castelli A, et al. Effects of Prone Position on Lung Recruitment and Ventilation-Perfusion Matching in Patients With COVID-19 Acute Respiratory Distress Syndrome: A Combined CT Scan/Electrical Impedance Tomography Study. Critical Care Medicine. 2022;50:723–32.

2. Wang Y, Zhong M, Dong M, Song J, Zheng Y, Wu W, et al. Prone positioning improves ventilation–perfusion matching assessed by electrical impedance tomography in patients with ARDS: a prospective physiological study. Crit Care. 2022;26:154.

3. Ling S, Zhao Z. End-Expiratory Lung Impedance Measured With Electrical Impedance Tomography at Different Days Are Not Comparable. Critical Care Medicine. 2023;51:e70–2.

4. Franchineau G, Jonkman AH, Piquilloud L, Yoshida T, Costa E, Rozé H, et al. Electrical Impedance Tomography to Monitor Hypoxemic Respiratory Failure. Am J Respir Crit Care Med. 2023;rccm.202306-1118CI.

5. Abrams D, Bacchetta M, Brodie D. Recirculation in venovenous extracorporeal membrane oxygenation. ASAIO J. 2015;61:115–21.
